# Quality of Life After Liver Transplantation—Results from a Large European Multinational Survey

**DOI:** 10.3390/jcm15187027

**Published:** 2026-09-10

**Authors:** Speranta Iacob, Teresa Antonini, Maria Cristina Morelli, Sara Pasquato, Liz Schick, Heiner Wedemeyer, Fabien Zoulim, Giacomo Germani

**Affiliations:** 1 Department of Gastroenterology and Hepatology, Faculty of Medicine, Carol Davila University of Medicine and Pharmacy, 020021 Bucharest, Romania; 2 Center of Digestive Diseases and Liver Transplantation, Fundeni Clinical Institute, 022328 Bucharest, Romania; 3 Center of Excellence in Translational Medicine, Fundeni Clinical Institute, 022328 Bucharest, Romania; 4 Hepatology Department, Hospices Civils de Lyon, University of Lyon, 69004 Lyon, France; teresa.antonini@chu-lyon.fr (T.A.); fabien.zoulim@inserm.fr (F.Z.); 5 Internal Medicine Unit for the Treatment of Severe Organ Failure, IRCCS Azienda Ospedaliero-Universitaria di Bologna, 40138 Bologna, Italy; mariacristina.morelli@aosp.bo.it; 6 Hospital Psychology Unit, Padua University Hospital, 35128 Padua, Italy; sara.pasquato@aopd.veneto.it; 7 World Transplant Games Federation, Winchester SO23 0LD, UK; liz.schick@wtgf.org; 8 Department of Gastroenterology, Hepatology, Infectious Diseases and Endocrinology, Hannover Medical School, 30625 Hannover, Germany; wedemeyer.heiner@mh-hannover.de; 9 Multivisceral Transplant Unit, Department of Surgery, Oncology and Gastroenterology, Azienda Ospedale-Università Padova, 35125 Padua, Italy; germani.giacomo@gmail.com

**Keywords:** liver transplantation, quality of life, patient-reported outcomes, European survey, ESOT questionnaire, cross-sectional survey

## Abstract

**Background/Objectives:** Liver transplantation (LT) is life-saving, yet its long-term impact on quality of life (QoL) remains complex and incompletely characterized. A standardized, transplant-specific patient-reported outcome measure (PROM) for routine use in European LT recipients is currently lacking. **Methods:** This descriptive, cross-sectional survey assessed QoL among 794 adult LT recipients across 22 European countries using a purpose-built, 48-item transplant-specific questionnaire developed within the European Society for Organ Transplantation (ESOT) Patient Inclusion Initiative. The instrument has not undergone psychometric validation and was used as an exploratory research survey. Respondents were grouped by time since LT (0–5, 6–12, and ≥13 years). Group comparisons used chi-square tests with Benjamini–Hochberg correction, followed by multivariable analyses adjusted for relevant sociodemographic factors. **Results:** Median time since LT was 6 years (IQR 3–12). After multiple-testing correction, alcohol consumption (q < 0.001), feeling that life is worth living (q = 0.029), and changes in social life (q = 0.029) differed between groups. In multivariable analysis, only alcohol consumption remained independently associated with time since LT, with progressively higher odds at 6–12 years (aOR 4.18, 95% CI 2.56–6.82) and ≥13 years (aOR 7.09, 95% CI 4.27–11.77) compared with 0–5 years. Physical health, emotional functioning, sexual health, satisfaction with care and medication adherence did not differ between groups. **Conclusions:** This large European survey provides hypothesis-generating information on self-reported well-being after LT. Alcohol consumption was the only outcome independently associated with time since transplantation. These findings support prospective studies using psychometrically validated patient-reported measures.

## 1. Introduction

Liver transplantation (LT) has advanced significantly over the years, particularly with the development of immunosuppressive therapies that have resulted in excellent short- and long-term survival rates. These advancements persist despite an aging recipient population, associated comorbidities, and a growing number of oncology cases among transplant patients [1,2,3,4]. Assessing quality of life (QoL) after LT has become essential in evaluating the success of this complex, life-saving procedure [5]. Beyond clinical and survival metrics, QoL captures the complex experiences of LT recipients, addressing their physical, psychological, and social well-being. The transplantation journey profoundly transforms patients’ lives, demanding adaptation to physical recovery, emotional resilience and societal reintegration. Understanding QoL outcomes enriches patient-centered care and provides physicians with critical insights to optimize long-term outcomes and address unmet needs [5,6,7]. This shift is reflected at the level of health policy: the ESOT Consensus Statement on Outcome Measures in Liver Transplantation advocates a move towards value-based health care, in which patient-reported outcomes are collected alongside graft and patient survival as indicators of transplant success [8]. Implementing such a framework across Europe presupposes instruments that are acceptable to patients, feasible in routine practice and specific enough to capture concerns unique to transplantation. Current literature highlights that while LT generally improves QoL, the extent of improvement differs across physical, mental and social domains. A foundational meta-analysis of 218 studies across 23 countries, involving 14,750 patients, demonstrated significant QoL improvements post-transplant compared to pretransplant levels and other chronic conditions [9]. Similarly, Bravata et al. [10] synthesized data from 49 studies involving 3576 LT recipients, reporting amelioration in all QoL domains, with the greatest gains in physical functioning and smaller improvements in psychosocial areas. Additionally, Nickel et al. [11] highlighted a decrease in negative mood states and an increase in vigor, reinforcing the emotional benefits of transplantation. Most of these data derive from single-centre or single-country cohorts assessed with generic instruments, so that domains of particular relevance to transplant recipients such as immunosuppression-related side effects, fear of rejection, body image, return to work, alcohol-related concerns and long-term medical surveillance are either absent or only indirectly addressed.

Despite these findings, a review by Jay et al. [12] highlighted the lack of rigorous, standardized QoL assessments for LT recipients, with most studies relying on generic health assessment tools. This observation has recently been reinforced by a systematic review [13] which comprehensively evaluated measurement properties of all available patient-reported outcome measures (PROMs) used in adult LT candidates and recipients and concluded that although the quality of evidence for most instruments remains low, the post-Liver Transplant Quality of Life (pLTQ) questionnaire demonstrates high-quality evidence for internal consistency, reliability, and responsiveness [13]; nevertheless, further validation and refinement of transplant-specific PROMs across varied post-LT populations remain warranted. Addressing this remaining methodological gap, namely, the need for further validation and refinement of transplant-specific PROMs across heterogeneous post-LT populations requires two sequential steps: an exploratory field-testing phase in a large and culturally diverse sample, to establish which items are understood, answered and discriminative; and a formal psychometric validation study designed according to COSMIN standards [14]. The present work belongs to the first of these two steps.

This study presents findings from a large European descriptive survey using a purpose-built, LT-specific questionnaire developed under the guidance of the European Society for Organ Transplantation (ESOT) Patient Inclusion Initiative. The questionnaire was constructed specifically to capture the multidimensional challenges of post-transplant life and was used here as a survey instrument in a large, multinational cohort. Our main objective was to assess the general well-being of LT recipients as a measure of success of this procedure and to emphasize the importance of discussion and support from dedicated transplant healthcare professionals across European regions for this group of patients. The specific objectives were: (i) to describe the demographic and self-reported well-being profile of adult LT recipients responding to a pan-European online survey; (ii) to examine whether item responses differ between groups defined by time since transplantation, with adjustment for the principal demographic confounders; and (iii) to evaluate the feasibility and item-level performance of the instrument in order to inform the design of a subsequent prospective validation study. No causal or longitudinal inference was intended, and none is possible from the present design.

## 2. Materials and Methods

### 2.1. Design

This was a descriptive, cross-sectional observational study based on a purpose-built survey questionnaire designed to assess multiple domains of well-being among liver transplant (LT) recipients from multiple European transplant centers. Reporting follows the STROBE statement for cross-sectional studies [15] (Supplementary File S2) and, for the internet-based components, the CHERRIES recommendations for reporting web surveys [16]. Because the design is cross-sectional and each participant contributed a single set of responses, all comparisons between post-transplant duration groups are between-subject comparisons and cannot be interpreted as within-patient change over time.

### 2.2. Participants

The study included 794 post-liver transplant adult recipients at the time of survey completion. Participants were recruited through convenience sampling during the study period (16 April–7 May 2024). Eligibility criteria were: age ≥18 years, previous liver transplantation, and completion of the post-transplant section of the questionnaire in one of the six available languages. Patients who had multiorgan or other solid organ transplants, severe mental illnesses, or were unable to respond were excluded.

**Figure 1:**
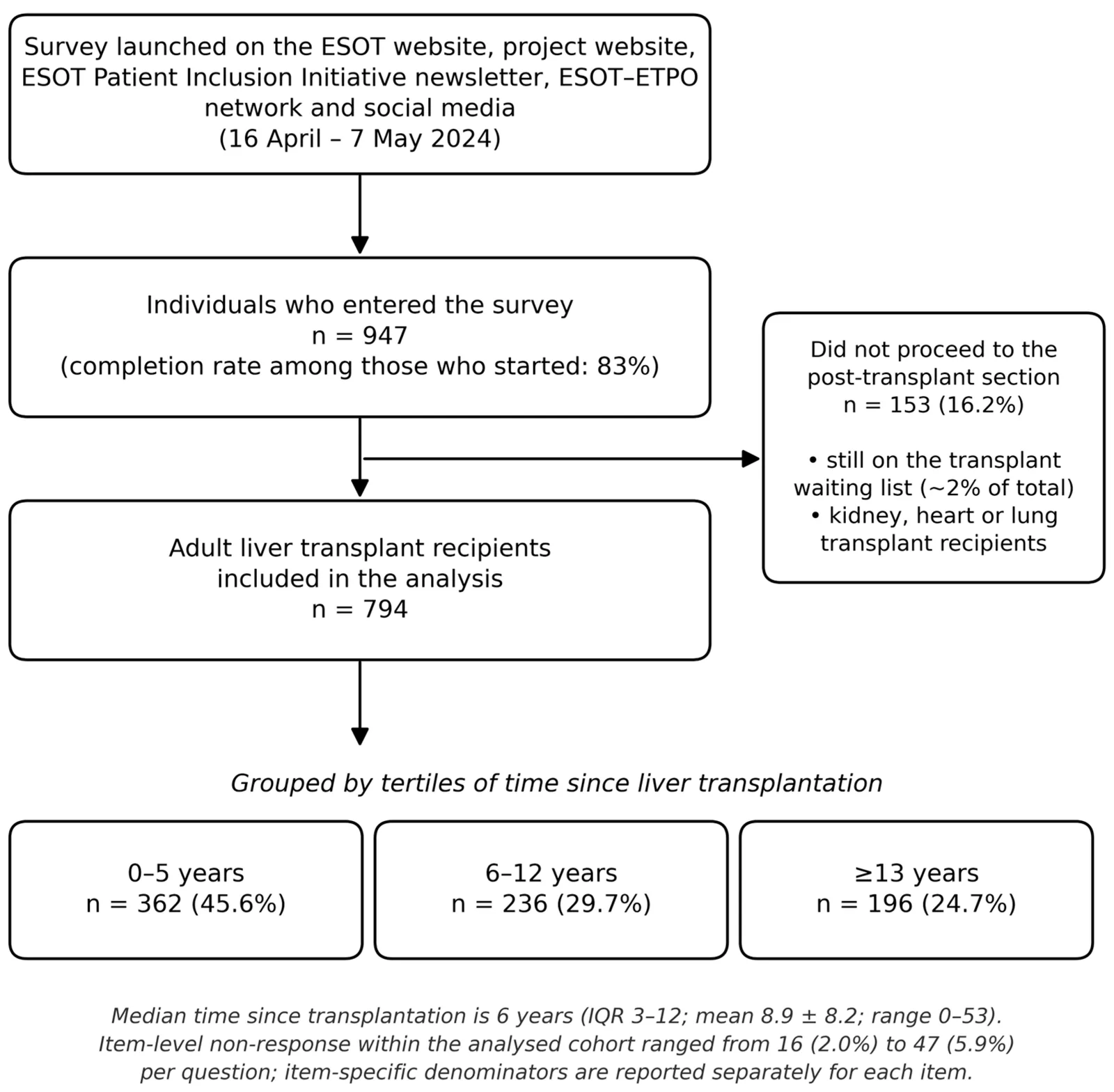
Flow of participants through the survey.

A total of 947 individuals entered the broader survey. For the present analysis, which was specifically restricted to adult liver transplant recipients, respondents who were on the transplant waiting list or had received kidney, heart or lung transplants were not included, resulting in a final analytic cohort of 794 liver transplant recipients (Figure 1). Consistent with the CHERRIES recommendations for reporting internet surveys [16], the completion rate among individuals who initiated the questionnaire was 83%. Item-level non-response within the analysed cohort was low and relatively uniform, ranging from 16 (2.0%) to 47 (5.9%) per question, and item-specific denominators are reported throughout. The survey was administered through the SurveyMonkey platform (SurveyMonkey Inc., San Mateo, CA, USA). Dataset-level screening across the available demographic, transplant-related and questionnaire variables identified no exact duplicate records.

### 2.3. Survey Questionnaire

The questionnaire used in this study is a transplant-specific survey instrument developed within the ESOT Patient Inclusion Initiative by an experienced clinical psychologist. Item development drew on established conceptual frameworks and adapted selected items from validated instruments (Short Form Health Survey SF-36, Nottingham Health Profile, Sickness Impact Profile) to the specific concerns of LT recipients, while incorporating new, transplant-specific items to address domains not covered by existing generic tools. The final instrument includes 48 items covering the following domains:

- Physical health and functioning;
- Emotional and psychological well-being;
- Social relationships and support;
- Lifestyle and habits;
- Role and occupational functioning;
- Sexual health;
- Perception of medical care;
- Post-transplant changes.

Most items use three-level ordinal response options (“not at all”/“yes, a little”/“yes, a lot”), with frequency-based response options for behavioural items and four-level response options for items related to medical care. The instrument comprises parallel sections for patients on the waiting list and for transplant recipients; the present analysis includes the post-transplant section, which is provided as Supplementary File S1. The present study is conceived as a pilot, hypothesis-generating investigation; its primary purpose is to characterize the scope and distribution of post-transplant QoL challenges across a large European cohort and to establish the empirical foundation required to design a methodologically rigorous follow-up study. Formal psychometric validation in an independent prospective cohort represents the next stage of instrument development, with the current dataset informing subsequent sampling and study design.

The self-administered questionnaire was available in several European languages (English, French, German, Italian, Romanian and Spanish), ensuring accessibility for participants from diverse linguistic backgrounds.

The survey was disseminated through the following methods: (1) launch on the ESOT website; (2) direct link distribution via the project website; (3) inclusion in the ESOT Patient Inclusion Initiative newsletter (20 April 2024); (4) targeted messaging to ESOT–ETPO network contacts; and (5) a social media campaign. These strategies ensured broad visibility and engagement across Europe. This dissemination strategy maximised geographical reach but, by design, could only reach patients connected to transplant societies, patient organisations or social media.

### 2.4. Definition of Post-Transplant Duration Groups

Time since transplantation was recorded in completed years. Given the distribution of post-transplant duration in the study population, respondents were categorized into approximate tertiles: 0–5 years (n = 362), 6–12 years (n = 236), and ≥13 years (n = 196) since LT. These categories were used for all between-group comparisons.

### 2.5. Ethical Considerations

All participants provided informed consent prior to participation by voluntarily completing the online questionnaire, which included full information on the purpose of the study, data protection, and GDPR compliance. As the study was observational, non-interventional and analysed anonymously, with no direct identifiers held in the analysis dataset and with no collection of sensitive personal data, no formal ethical approval was required according to ESOT policy. The study was conducted in accordance with the Declaration of Helsinki.

### 2.6. Statistical Analysis

Data were recorded in a Microsoft^®^ Office Excel spreadsheet and statistical analysis was performed using NCSS (version 23.0.2, NCSS, LLC, Kaysville, UT, USA). Categorical variables are presented as frequencies (percentages) and continuous variables as mean ± standard deviation (SD) and as median with interquartile range (IQR) where the distribution was skewed. Percentages for categorical variables were calculated within each post-transplant duration group, using the number of respondents who answered the corresponding item as the denominator. Missing responses were reported separately and were not imputed. Differences between categorical variables were analyzed using the chi-square test; no composite domain scores were computed, and analyses are limited to descriptive and group-comparison levels consistent with the exploratory, field-testing nature of this survey. Because 20 item-level comparisons were performed across post-transplant duration groups, the Benjamini–Hochberg procedure was applied to control the false discovery rate at q = 0.05 [17]. Unadjusted *p*-values and adjusted q-values are reported side by side. For items showing nominal significance in univariate analysis, multivariable binary logistic regression models were fitted, adjusting for post-transplant duration group, age band, sex, European region, education and employment status. Results are presented as adjusted odds ratios (aOR) with 95% confidence intervals. A two-tailed *p*-value < 0.05 was considered statistically significant.

## 3. Results

This study analyzed data from 794 LT recipients across 22 countries, with a mean post-transplant duration of 8.9 ± 8.2 years (median 6 years, IQR 3–12; range: 0–53 years). Median age at transplantation was 52 years (IQR 42–60; mean 49.1 ± 16.3). Participant flow is shown in Figure 1. Table 1 provides a detailed breakdown of sociodemographic characteristics.

Age distributions revealed a predominance of older age groups, with patients aged 61–70 forming the largest segment, particularly in Western Europe (37.34%) and Southern Europe (32.26%). The 51–60 age group was also notably represented, especially in Eastern Europe (30.10%). A Chi-Square test confirmed significant differences in age distribution across regions (*p* < 0.0001).

To explore whether self-reported well-being differs according to time elapsed since transplantation, recipients were divided into three groups based on time since LT: 0–5 years (362 patients, 45.6%), 6–12 years (236 patients, 29.7%), and ≥13 years (196 patients, 24.7%).

### 3.1. Demographics and Regional Variations

The regional distribution of LT recipients varied significantly between groups (*p* = 0.02). Western Europe consistently accounted for the largest proportion in all groups, peaking in the ≥13-year group (82.7%). Eastern Europe had a higher representation among respondents 0–5 years (14.9%) and 6–12 years (16.1%) after transplantation and was less represented in the ≥13-year group (5.6%).

### 3.2. Age and Employment Trends

Younger patients (<40 years) were more frequently represented among respondents assessed within five years of transplantation (4.4% aged 18–30; 11.9% aged 31–40; *p* < 0.0001). Older patients (>70 years) were more frequent among respondents assessed longest after transplantation (36.7% in the ≥13 year group vs. 4.1% in the 0–5 year group). Employment differed significantly between groups (*p* = 0.002): among respondents 0–5 years after LT, 25.0% were employed and 19.1% were professionals. The proportion of retired individuals was higher in the groups with longer time since LT, reaching 61.8% in the ≥13 year group. This gradient largely mirrors the age structure of the groups and is the reason why the item-level comparisons were subsequently adjusted for age and employment status (Section 3.8).

### 3.3. Physical Health and Functioning

Poor sleep at night was the only item within the physical and psychological domains to reach nominal significance (*p* = 0.035; q = 0.139). In the 0–5 year group, 41.0% reported mild sleep problems. The 6–12 year group had the highest proportion reporting no sleep problems (55.3%). In the ≥13 year group, mild sleep problems were reported by 43.9%. After correction for multiple testing, the between-group difference did not remain statistically significant and is therefore presented as exploratory. Sleep disturbance itself remained common across the cohort, with 50.2% of respondents reporting some degree of poor sleep at night (38.9% “a little”, 11.3% “a lot”). Other physical health measures, including general health perception, physical activity limitations and pain did not differ significantly between groups (Table 2).

### 3.4. Emotional and Psychological Well-Being

Most psychosocial parameters, including nervousness, happiness, and anxiety management, did not differ between groups. However, the perception of life being “worthy” differed significantly (*p* = 0.004; q = 0.029): the intermediate group (6–12 years) reported the lowest levels (80.3% responding “yes, a lot”), compared to 87.6% in the ≥13-year group. The cross-sectional design does not allow any inference about change within individuals, and this difference did not persist after multivariable adjustment (Section 3.8).

### 3.5. Social Relationships and Support

Self-isolation was less frequently reported in groups with longer time since LT (*p* = 0.017; q = 0.086): 75.4% of respondents 0–5 years after LT reported “not at all isolated,” compared to 82.9% in the ≥13-year group; this difference did not survive correction for multiple testing. Respondents assessed within five years of transplantation more frequently reported major social life changes (“yes, a lot” at 28.4%), while respondents assessed thirteen or more years after transplantation more frequently reported no change (49.7%; *p* = 0.004; q = 0.029).

### 3.6. Lifestyle and Habits

Respondents 0–5 years after LT showed higher rates of alcohol abstinence (53.7% never drank; 37.5% stopped post-transplant). Conversely, respondents ≥13 years after LT had higher proportions of occasional alcohol consumption “less than once a week” (23.7%) and “once a week” (11.6%) (*p* < 0.0001; q < 0.001). Smoking, unhealthy eating, and physical activity levels did not differ significantly across groups. Sexual health (*p* = 0.64) and changes in physical appearance (*p* = 0.25) also did not differ between groups. Neither the quantity of alcohol consumed nor the indication for transplantation was recorded, so these figures describe self-reported drinking frequency only and cannot be equated with relapse into harmful use. A separate item on behavioural change after transplantation was answered concordantly: 41.2% of respondents reported having changed their drinking habits, 42.7% had never drunk alcohol and 16.1% reported no change, closely matching the distribution of the frequency item.

### 3.7. Perception of Medical Care

The perception of receiving complete information about LT did not differ between groups (*p* = 0.15): 80.0% of recipients across all groups reported receiving complete information, with high satisfaction (76.1% completely satisfied; *p* = 0.56) and high medication adherence (“always” adhering: 88.1%; *p* = 0.232).

Despite this, a final item asking which topics respondents would like more information about revealed substantial unmet needs (n = 747). Most frequently endorsed were life expectancy after LT (63.0%), health limitations after transplantation (56.4%) and the side effects of immunosuppressive therapy (53.5%); 45.6% wished for more information on sport activity, 42.7% on the impact of transplantation on sexual life and 39.6% on its impact on working activity (Table 3).

### 3.8. Multivariable Analysis

**Figure 2:**
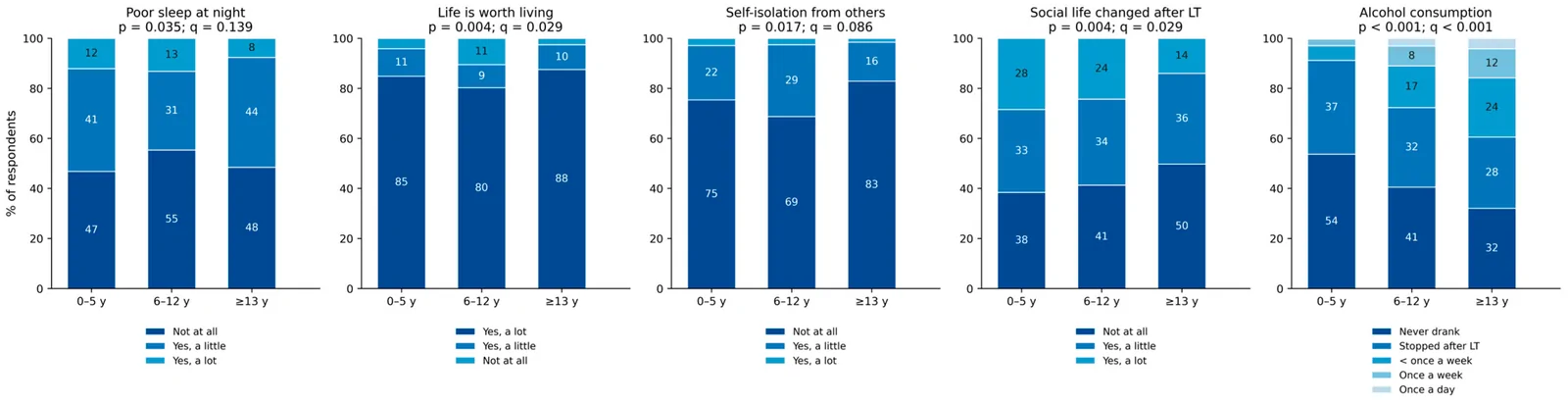
Distribution of responses to the five items of principal interest, by time since liver transplantation.

Multivariable models adjusted for age band, sex, European region, education and employment status are presented in Table 4. The association between time since transplantation and alcohol consumption remained independently significant. Compared with the 0–5-year group, the adjusted odds of current alcohol intake were 4.18 (95% CI 2.56–6.82; *p* < 0.001) at 6–12 years and 7.09 (95% CI 4.27–11.77; *p* < 0.001) at ≥13 years. Higher education was also independently associated with current alcohol intake (aOR 1.60, 95% CI 1.10–2.32; *p* = 0.014). Neither the feeling that life is worth living nor self-reported change in social life remained independently associated with time since transplantation after adjustment. Age was independently associated with change in social life (aOR 0.84 per age-band increase, 95% CI 0.73–0.97; *p* = 0.021). Self-isolation was less frequent in the ≥13-year group (aOR 0.61, 95% CI 0.38–0.98; *p* = 0.041) and among currently working respondents (aOR 0.59, 95% CI 0.38–0.93; *p* = 0.023). Sleep problems were less frequent in the 6–12-year group (aOR 0.67, 95% CI 0.48–0.95; *p* = 0.024), in men (aOR 0.68, 95% CI 0.50–0.91; *p* = 0.009), and among respondents with higher education (aOR 0.74, 95% CI 0.55–0.98; *p* = 0.039) (Figure 2).

## 4. Discussion

LT is universally acknowledged as a life-saving intervention, yet its long-term impact on recipients’ QoL remains a complex and evolving challenge. After correction for multiple testing and multivariable adjustment, alcohol consumption emerged as the clearest association with time since transplantation, with progressively higher self-reported alcohol intake in groups further from transplantation. Other between-group differences observed in the univariate analysis were attenuated after adjustment, highlighting the potential influence of demographic characteristics, particularly age, when interpreting QoL across different post-transplant periods. Given the cross-sectional design, differences between post-transplant duration groups should be interpreted as associations rather than within-individual changes over time and may also reflect cohort and survivorship effects. Echoing concerns raised by Burra and Germani [5], who emphasized that QoL gains tend to wane after the first post-transplant year and vary widely across individual domains, our findings reveal differences in selected aspects of physical, emotional, and social well-being between post-transplant duration groups.

Among LT recipients, 11.7% reported their overall health as excellent, 31.5% as very good, 37.3% as good, 15.3% as fair, and 4.2% as poor. Although this distribution might appear modest compared to general population norms [18], it becomes more meaningful when adjusted for age structure. As our cohort predominantly comprises individuals over 50 years of age, especially in the 61–70 intervals in Western and Southern Europe, these results are encouraging when compared with age-matched population data from published surveys [19,20]. Since age strongly correlates with declines in self-reported health [7], it is not surprising that LT recipients predominantly older, report somewhat lower levels of perceived health. Our findings suggest that their health perceptions (general health, physical limitations, pain, nervousness, managing worry) are in line with age-matched peers in some domains [21,22,23] and in others (happiness, physical activity) may even exceed those of non-transplanted individuals with chronic illnesses of similar age.

### 4.1. Physical Health and Functioning

Poor sleep at night was the only item in the physical and psychological domains to reach nominal significance, but it did not remain significant after correction for multiple testing; the following interpretation is therefore exploratory. This association may reflect differences in clinical characteristics, medication exposure, and other factors known to influence sleep quality among transplant recipients at different stages after transplantation [24]. Sleep nonetheless deserves attention because of its prevalence rather than any between-group difference: half of all respondents reported some degree of poor sleep. This is consistent with recent multicentre data in which insomnia was significantly more frequent among LT recipients surviving beyond twenty years than in age-matched controls (32% vs. 12%, *p* = 0.03) [25], supporting sleep as a domain warranting dedicated assessment in future studies.

Other physical domains, including general health perception, physical activity limitations and pain did not differ significantly between groups, though the absolute proportion of patients reporting pain or limitations remains notable in comparison to the general population. A study assessing LT recipients beyond 60 years using the SF-36 found significantly lower scores in physical functioning, bodily pain, and general health compared to age-matched general population controls [7]. This underscores that while LT recipients achieve satisfactory QoL overall, physical domains generally remain below those of the general population in older adults, and targeted physical rehabilitation programs are needed [26,27].

### 4.2. Emotional and Psychological Well-Being

Most psychosocial parameters did not differ between groups. The lower proportion of respondents endorsing life-worth in the 6–12-year group, compared with both the 0–5-year and the ≥13-year groups, is consistent with reports of variable psychosocial adjustment after liver transplantation [28,29]. Because patients were assessed at a single time point, this difference reflects a comparison between groups rather than change within individuals, and may relate to concerns that are more prominent at this stage of follow-up. The higher endorsement in the ≥13-year group may be compatible with elements of posttraumatic growth (PTG), an increasingly recognized concept in transplant survivorship [30,31,32,33]. In a Spanish cohort, Funuyet-Salas et al. [30] found that LT recipients with better self-perceived health and higher vitality reported significantly greater PTG, especially in appreciation of life, new possibilities, and personal strength. PTG was not directly assessed in the present survey, and the observed difference did not persist after multivariable adjustment. A survivorship effect may also contribute to this pattern and should be considered when interpreting the findings.

### 4.3. Social Relationships and Support

Self-isolation was less frequent and self-reported change in social life less pronounced, among respondents assessed longer after transplantation. This aligns with prior literature [34,35] describing better reestablishment of social roles, driven by improved physical recovery, self-confidence, and diminished stigma. In multivariable analysis, however, the difference in social life was explained by age rather than by time since transplantation, and only self-isolation retained an independent association. Our findings reinforce the importance of long-term psychosocial support, especially in the first year post-transplant [36].

### 4.4. Lifestyle and Habits

The difference in alcohol consumption between groups was the most robust finding of this survey, being the only association to survive both correction for multiple testing and multivariable adjustment: while the majority of respondents assessed within five years of transplantation maintained complete abstinence, a proportion of over 30% of respondents assessed thirteen or more years after LT reported occasional alcohol use. This trend, statistically significant at *p* < 0.0001, is consistent with published literature reporting that up to 30–50% of LT recipients consume some alcohol within five years post-transplant, often at low or occasional levels [37,38]. A separate item on behavioural change was answered concordantly, which argues against careless responding on this topic. The questionnaire assessed frequency, but not quantity, of alcohol intake and did not record indication for transplantation; therefore, these findings should be interpreted as patterns of self-reported alcohol consumption rather than relapse or harmful use. Despite these limitations, the association with time since transplantation remained significant after correction for multiple testing and multivariable adjustment, supporting continued attention to alcohol use during long-term follow-up.

These findings reinforce the importance of ongoing psychosocial monitoring. Smoking did not differ between groups; 75.9% of respondents had never smoked and 17.5% had stopped after transplantation, proportions that compare favourably with published post-transplant cohorts, in which continued smoking is common and associated with adverse outcomes [39,40]. Regarding sexual health and body image, the stable figures across groups suggest that psychosocial adjustment is comparable across groups. Two findings are notable for their magnitude rather than for any between-group difference: only 34.2% of respondents were fully satisfied with their sexual life and 28.1% reported no satisfaction at all, and 42.7% wished for more information about the impact of transplantation on sexual life. Neither varied between groups, which suggests these are not transient consequences of surgery and early immunosuppression but persistent features of life after LT. The persistent challenges related to body image and sexual health, often linked to medication side effects [41], underline the importance of incorporating psychosexual support as an integral component of multidisciplinary post-transplant care [42].

### 4.5. Perception of Medical Care

High levels of satisfaction with information, consistency in perceived care quality, and high medication adherence across all time groups are clinically meaningful findings. Multiple studies [43,44] confirm that the majority of LT recipients feel well-informed, and higher satisfaction is linked to better adherence. Adherence to immunosuppression in adult LT recipients ranges from 60–85%, depending on measurement methods, with nonadherence rates of 15–40% [45]. The high self-reported medication adherence (88.1%) should be interpreted in the context of the survey population and recruitment strategy. An important finding emerges when satisfaction with information is considered alongside expressed needs. Although 80.0% of respondents reported having received complete information about transplantation, 63.0% wished for more information about life expectancy after LT, 56.4% about health limitations, and 53.5% about the side effects of immunosuppressive therapy. These findings suggest that patient information needs persist beyond the peri-transplant period and support ongoing education as an integral component of long-term follow-up. This is aligned with the ESOT Consensus on Outcomes Measures in Liver Transplantation, which advocates incorporating patient-reported outcomes alongside clinical measures within a Value-Based Health Care framework [8].

### 4.6. Strengths and Limitations

This study has several important strengths. It includes a large and geographically diverse cohort of 794 liver transplant recipients from 22 European countries, encompassing a broad range of post-transplant durations (0–53 years). The multilingual survey captures the patient perspective across multiple dimensions of post-transplant life, including physical, psychological, social, behavioural and healthcare-related aspects. The statistical approach, incorporating correction for multiple testing and multivariable adjustment, strengthens the interpretation of the observed between-group differences. Importantly, the study also identifies clinically relevant areas deserving continued attention, particularly alcohol consumption, sexual health and persistent information needs, providing a valuable basis for future prospective patient-centred research after LT.

We also acknowledge the limitations of the study. The cross-sectional nature limits our ability to assess causality or capture individual QoL change over time; the three groups comprise different individuals, and observed differences may reflect cohort and survivorship effects as much as post-transplant adaptation. Second, the post-transplant duration groups were based on the distribution of time since transplantation, with relatively few respondents represented in the first post-transplant year. Third, the convenience sampling approach and online dissemination method may have introduced selection bias, as respondents with greater health literacy or internet access may have been over-represented. Nevertheless, the survey achieved an 83% completion rate, with low item-level non-response, supporting good response completeness within the analysed cohort. Fourth, the questionnaire has not yet undergone formal psychometric validation and is therefore used here as an exploratory, hypothesis-generating instrument. Formal validation in an independent prospective cohort represents the next stage of its development. Finally, detailed clinical variables, including indication for transplantation, comorbidities, immunosuppressive regimen and graft-related outcomes, were not collected within this anonymous patient-reported survey. Their integration with patient-reported outcomes will be important in future prospective studies. As with all questionnaire-based studies, the findings rely on self-reported information.

This work responds to the gap highlighted by the COSMIN review by van Knippenberg et al. [13], which found low-quality evidence for most existing PROMs in adult liver transplant populations and identified the need for further validation across culturally and organizationally diverse European post-LT populations. The present dataset forms the basis for a planned prospective validation study, including refinement of domain structure, optimisation of item performance and determination of sample size for confirmatory psychometric analyses within recognised PROM development frameworks [14].

## 5. Conclusions

This large European cross-sectional survey provides a broad patient-reported perspective on well-being after liver transplantation across 22 countries. Overall, most physical, psychological, social and healthcare-related outcomes were comparable across post-transplant duration groups, while alcohol consumption showed a strong and independent association with time since transplantation. The survey also identified persistent needs related to sexual health and information on life expectancy, functional limitations and treatment-related effects, highlighting aspects of post-transplant life that extend beyond conventional clinical outcomes. These findings reinforce the importance of a patient-centred approach to long-term post-transplant care and provide a strong foundation for prospective studies integrating validated patient-reported measures with clinical data.

## Figures and Tables

**Table 1:** Sociodemographic factors of study participants (N = 794), overall and by time since liver transplantation. Data are n (%); percentages are calculated within columns among respondents who answered the item.

Sociodemographic Profile	0–5 y (n = 362)	6–12 y (n = 236)	≥13 y (n = 196)	Total (N = 794)	*p*
**Region**					0.021
Western Europe	275 (76.0%)	171 (72.5%)	162 (82.7%)	608 (76.6%)	
Southern Europe	24 (6.6%)	19 (8.1%)	19 (9.7%)	62 (7.8%)	
Eastern Europe	54 (14.9%)	38 (16.1%)	11 (5.6%)	103 (13.0%)	
Non-EU countries	9 (2.5%)	8 (3.4%)	4 (2.0%)	21 (2.6%)	
**Gender** (8 not reported)					0.97
Male	195 (54.0%)	125 (53.2%)	103 (54.2%)	423 (53.8%)	
Female	166 (46.0%)	110 (46.8%)	87 (45.8%)	363 (46.2%)	
**Age groups**					<0.0001
18–30 years	16 (4.4%)	13 (5.5%)	4 (2.0%)	33 (4.2%)	
31–40 years	43 (11.9%)	17 (7.2%)	7 (3.6%)	67 (8.4%)	
41–50 years	54 (14.9%)	18 (7.6%)	12 (6.1%)	84 (10.6%)	
51–60 years	106 (29.3%)	65 (27.5%)	43 (21.9%)	214 (27.0%)	
61–70 years	128 (35.4%)	94 (39.8%)	58 (29.6%)	280 (35.3%)	
71–85 years	15 (4.1%)	29 (12.3%)	72 (36.7%)	116 (14.6%)	
**Age at transplantation, mean ± SD**	50.7 ± 15.5	49.5 ± 16.5	45.7 ± 17.0	49.1 ± 16.3	<0.01
**Level of education**					0.35
Primary School	7 (1.9%)	8 (3.4%)	10 (5.1%)	25 (3.1%)	
Middle School	68 (18.8%)	33 (14.0%)	25 (12.8%)	126 (15.9%)	
High School	110 (30.4%)	79 (33.5%)	71 (36.2%)	260 (32.7%)	
Bachelor	95 (26.2%)	65 (27.5%)	43 (21.9%)	203 (25.6%)	
Master	65 (18.0%)	42 (17.8%)	37 (18.9%)	144 (18.1%)	
PhD	17 (4.7%)	9 (3.8%)	10 (5.1%)	36 (4.5%)	
**Occupation** (**n = 608**)					0.003
Employee	64 (25.0%)	34 (18.2%)	25 (15.2%)	123 (20.2%)	
Professional	49 (19.1%)	20 (10.7%)	22 (13.3%)	91 (15.0%)	
Service/Trade worker	9 (3.5%)	3 (1.6%)	1 (0.6%)	13 (2.1%)	
Farm/Artisan/Factory worker	5 (2.0%)	3 (1.6%)	1 (0.6%)	9 (1.5%)	
Houseworker	5 (2.0%)	6 (3.2%)	3 (1.8%)	14 (2.3%)	
Student	2 (0.8%)	4 (2.1%)	3 (1.8%)	9 (1.5%)	
Unemployed	21 (8.2%)	10 (5.3%)	8 (4.8%)	39 (6.4%)	
Retired	101 (39.5%)	107 (57.2%)	102 (61.8%)	310 (51.0%)	
**Marital status**					0.50
Married	249 (68.8%)	142 (60.2%)	129 (65.8%)	520 (65.5%)	
Single	43 (11.9%)	38 (16.1%)	24 (12.2%)	105 (13.2%)	
Unmarried partner	30 (8.3%)	22 (9.3%)	16 (8.2%)	68 (8.6%)	
Divorced	24 (6.6%)	14 (5.9%)	11 (5.6%)	49 (6.2%)	
Separated	5 (1.4%)	6 (2.5%)	2 (1.0%)	13 (1.6%)	
Widowed	8 (2.2%)	11 (4.7%)	10 (5.1%)	29 (3.7%)	
I prefer not to say	3 (0.8%)	3 (1.3%)	4 (2.0%)	10 (1.3%)	

**Table 2:** Patient-Reported Outcomes related to general health, behavior and emotional well-being by post-transplant duration. Data are n (%) with percentages calculated within each column; the denominator is the number of respondents in that group who answered the item. q = Benjamini–Hochberg adjusted *p*-value across the 20 comparisons. * Smoking item: single response allowed; intensity categories overlap and cannot be interpreted precisely.

Question	0–5 Years	6–12 Years	≥13 Years	Total	*p* Value	q Value
**Alcohol consumption (n = 756)**					<0.001	<0.001
I never drank alcohol	182 (53.7%)	92 (40.5%)	61 (32.1%)	335 (44.3%)		
I have stopped since my transplant	127 (37.5%)	72 (31.7%)	54 (28.4%)	253 (33.5%)		
Less than once a week	20 (5.9%)	38 (16.7%)	45 (23.7%)	103 (13.6%)		
Once a week	9 (2.7%)	18 (7.9%)	22 (11.6%)	49 (6.5%)		
Once a day	1 (0.3%)	7 (3.1%)	8 (4.2%)	16 (2.1%)		
**Feeling that life is worth living (n = 778)**					0.004	0.029
Yes, a lot	303 (84.9%)	183 (80.3%)	169 (87.6%)	655 (84.2%)		
Yes, a little	39 (10.9%)	21 (9.2%)	19 (9.8%)	79 (10.2%)		
Not at all	15 (4.2%)	24 (10.5%)	5 (2.6%)	44 (5.7%)		
**Social life changed after LT (n = 764)**					0.004	0.029
Not at all	131 (38.4%)	95 (41.3%)	96 (49.7%)	322 (42.1%)		
Yes, a little	113 (33.1%)	79 (34.3%)	70 (36.3%)	262 (34.3%)		
Yes, a lot	97 (28.4%)	56 (24.3%)	27 (14.0%)	180 (23.6%)		
**Self-isolation from others (n = 764)**					0.017	0.086
Not at all	257 (75.4%)	158 (68.7%)	160 (82.9%)	575 (75.3%)		
Yes, a little	74 (21.7%)	66 (28.7%)	30 (15.5%)	170 (22.3%)		
Yes, a lot	10 (2.9%)	6 (2.6%)	3 (1.6%)	19 (2.5%)		
**Poor sleep at night (n = 777)**					0.035	0.139
Not at all	162 (46.8%)	130 (55.3%)	95 (48.5%)	387 (49.8%)		
Yes, a little	142 (41.0%)	74 (31.5%)	86 (43.9%)	302 (38.9%)		
Yes, a lot	42 (12.1%)	31 (13.2%)	15 (7.7%)	88 (11.3%)		
**Received appropriate information about LT (n = 756)**					0.15	0.417
Yes, completely	275 (81.1%)	187 (82.4%)	143 (75.3%)	605 (80.0%)		
Yes, partially	54 (15.9%)	33 (14.5%)	32 (16.8%)	119 (15.7%)		
Very little	7 (2.1%)	4 (1.8%)	10 (5.3%)	21 (2.8%)		
Not at all	3 (0.9%)	3 (1.3%)	5 (2.6%)	11 (1.5%)		
**Feeling happy (n = 764)**					0.16	0.417
Yes, a lot	235 (68.9%)	152 (66.1%)	137 (71.0%)	524 (68.6%)		
Yes, a little	95 (27.9%)	62 (27.0%)	51 (26.4%)	208 (27.2%)		
Not at all	11 (3.2%)	16 (7.0%)	5 (2.6%)	32 (4.2%)		
**Sports activities frequency (n = 784)**					0.19	0.417
≥2 times/week	184 (51.7%)	101 (43.0%)	85 (44.0%)	370 (47.2%)		
At least once/week	58 (16.3%)	59 (25.1%)	48 (24.9%)	165 (21.0%)		
≥2 times/month	33 (9.3%)	27 (11.5%)	17 (8.8%)	77 (9.8%)		
At least once/month	24 (6.7%)	15 (6.4%)	13 (6.7%)	52 (6.6%)		
Less than once/month	57 (16.0%)	33 (14.0%)	30 (15.5%)	120 (15.3%)		
**Nervousness level (n = 764)**					0.20	0.417
Not at all	206 (60.4%)	140 (60.9%)	135 (69.9%)	481 (63.0%)		
Yes, a little	115 (33.7%)	77 (33.5%)	52 (26.9%)	244 (31.9%)		
Yes, a lot	20 (5.9%)	13 (5.7%)	6 (3.1%)	39 (5.1%)		
**Changed perception about life (n = 756)**					0.23	0.417
Yes, a lot	211 (62.2%)	142 (62.6%)	105 (55.3%)	458 (60.6%)		
Yes, a little	106 (31.3%)	71 (31.3%)	64 (33.7%)	241 (31.9%)		
Not at all	22 (6.5%)	14 (6.2%)	21 (11.1%)	57 (7.5%)		
**Medication adherence (n = 756)**					0.232	0.417
Always	301 (88.8%)	197 (86.8%)	168 (88.4%)	666 (88.1%)		
Often	36 (10.6%)	24 (10.6%)	19 (10.0%)	79 (10.4%)		
**Sometimes**	1 (0.3%)	4 (1.8%)	0 (0.0%)	5 (0.7%)		
**Rarely**	0 (0.0%)	0 (0.0%)	1 (0.5%)	1 (0.1%)		
**Never**	1 (0.3%)	2 (0.9%)	2 (1.1%)	5 (0.7%)		
**Change in physical appearance perceived as positive (n = 756)**					0.25	0.417
Yes, completely	129 (38.1%)	83 (36.6%)	67 (35.3%)	279 (36.9%)		
Yes, partially	134 (39.5%)	80 (35.2%)	66 (34.7%)	280 (37.0%)		
Very little	44 (13.0%)	40 (17.6%)	27 (14.2%)	111 (14.7%)		
Not at all	32 (9.4%)	24 (10.6%)	30 (15.8%)	86 (11.4%)		
**Unhealthy eating behaviour (n = 756)**					0.33	0.514
Never ate more than I should or ate unhealthy food	91 (26.8%)	45 (19.8%)	49 (25.8%)	185 (24.5%)		
I have stopped since my transplant	23 (6.8%)	15 (6.6%)	7 (3.7%)	45 (6.0%)		
**Rarely**	84 (24.8%)	65 (28.6%)	56 (29.5%)	205 (27.1%)		
Occasionally	141 (41.6%)	102 (44.9%)	78 (41.1%)	321 (42.5%)		
* Smoking habit (n = 756)					0.39	0.559
I have never smoked cigarettes	261 (77.0%)	174 (76.7%)	139 (73.2%)	574 (75.9%)		
I have quit smoking since my transplant	51 (15.0%)	41 (18.1%)	40 (21.1%)	132 (17.5%)		
More than 5 in a day	12 (3.5%)	8 (3.5%)	7 (3.7%)	27 (3.6%)		
More than 10 in a day	12 (3.5%)	4 (1.8%)	4 (2.1%)	20 (2.6%)		
More than a packet in a day	3 (0.9%)	0 (0%)	0 (0%)	3 (0.4%)		
**Health-related limitations in daily physical activities (n = 777)**					0.47	0.623
Not at all	176 (50.9%)	134 (57.0%)	104 (53.1%)	414 (53.3%)		
Yes, a little	139 (40.2%)	80 (34.0%)	79 (40.3%)	298 (38.4%)		
Yes, a lot	31 (9.0%)	21 (8.9%)	13 (6.6%)	65 (8.4%)		
**Satisfaction with information on therapy (n = 756)**					0.56	0.696
Yes, completely	254 (74.9%)	175 (77.1%)	146 (76.8%)	575 (76.1%)		
Yes, partially	63 (18.6%)	38 (16.7%)	28 (14.7%)	129 (17.1%)		
Very little	15 (4.4%)	9 (4.0%)	14 (7.4%)	38 (5.0%)		
Not at all	7 (2.1%)	5 (2.2%)	2 (1.1%)	14 (1.9%)		
**Extent of perceived physical pain (n = 777)**					0.61	0.712
Not at all	204 (59.0%)	137 (58.3%)	126 (64.3%)	467 (60.1%)		
Yes, a little	125 (36.1%)	87 (37.0%)	59 (30.1%)	271 (34.9%)		
Yes, a lot	17 (4.9%)	11 (4.7%)	11 (5.6%)	39 (5.0%)		
**Satisfaction with sexual life (n = 764)**					0.65	0.712
Yes, a lot	122 (35.8%)	73 (31.7%)	66 (34.2%)	261 (34.2%)		
Yes, a little	119 (34.9%)	95 (41.3%)	74 (38.3%)	288 (37.7%)		
Not at all	100 (29.3%)	62 (27.0%)	53 (27.5%)	215 (28.1%)		
**Ability to manage worry (n = 764)**					0.68	0.712
Yes, a lot	146 (42.8%)	107 (46.5%)	92 (47.7%)	345 (45.2%)		
Yes, a little	156 (45.7%)	93 (40.4%)	78 (40.4%)	327 (42.8%)		
Not at all	39 (11.4%)	30 (13.0%)	23 (11.9%)	92 (12.0%)		
**Self-reported general health perception (n = 778)**					0.92	0.920
Excellent	40 (11.6%)	31 (13.1%)	20 (10.2%)	91 (11.7%)		
Very good	113 (32.7%)	71 (30.1%)	61 (31.1%)	245 (31.5%)		
Good	129 (37.3%)	85 (36.0%)	76 (38.8%)	290 (37.3%)		
Fair	47 (13.6%)	40 (16.9%)	32 (16.3%)	119 (15.3%)		
Poor	17 (4.9%)	9 (3.8%)	7 (3.6%)	33 (4.2%)		

**Table 3:** Topics on which respondents wished for more information (n = 747).

Topic	Yes, n (%)
Life expectancy after liver transplantation	469 (63.0%)
Health limitations after liver transplantation	417 (56.4%)
Side effects of the pharmacological therapies	394 (53.5%)
Impact of liver transplantation on regular sport activity	338 (45.6%)
Impact of liver transplantation on sex life	316 (42.7%)
Impact of liver transplantation on working activity	293 (39.6%)

**Table 4:** Multivariable logistic regression models. Adjusted odds ratios (95% CI). Dependent variables were dichotomised as follows: alcohol—any current intake (less than once a week, once a week, once a day) vs. never drank or stopped after transplantation; life worth living—“yes, a lot” vs. less; social life—any change vs. none; self-isolation—any vs. none; sleep—any problem vs. none. Reference categories: 0–5 years since LT, female sex, Western Europe, education below Bachelor’s degree, not currently working.

Covariate	Alcohol (n = 749)	Life Worth Living (n = 770)	Social Life Changed (n = 756)	Self-Isolation (n = 756)	Sleep Problem (n = 769)
6–12 y since LT	4.18 (2.56–6.82) *	0.77 (0.49–1.21)	0.91 (0.64–1.29)	1.37 (0.93–2.00)	0.67 (0.48–0.95) *
≥13 y since LT	7.09 (4.27–11.77) *	1.45 (0.84–2.50)	0.70 (0.48–1.02)	0.61 (0.38–0.98) *	0.87 (0.60–1.27)
Age band (per category)	1.09 (0.91–1.31)	0.86 (0.71–1.04)	0.84 (0.73–0.97) *	0.89 (0.76–1.04)	1.03 (0.90–1.18)
Male sex	1.18 (0.81–1.73)	1.30 (0.88–1.94)	0.81 (0.60–1.09)	0.94 (0.66–1.32)	0.68 (0.50–0.91) *
Bachelor’s degree or higher	1.60 (1.10–2.32) *	1.03 (0.69–1.52)	0.91 (0.67–1.22)	1.06 (0.75–1.49)	0.74 (0.55–0.98) *
Eastern Europe	0.59 (0.30–1.14)	1.36 (0.70–2.65)	0.69 (0.44–1.09)	0.59 (0.34–1.02)	0.87 (0.55–1.35)
Southern Europe	0.48 (0.21–1.07)	0.63 (0.33–1.20)	0.61 (0.36–1.06)	0.80 (0.42–1.54)	0.70 (0.41–1.21)
Non-EU country	0.50 (0.14–1.86)	0.92 (0.26–3.28)	3.47 (0.99–12.19)	2.46 (0.96–6.29)	2.07 (0.80–5.37)
Currently working	1.20 (0.72–1.98)	1.05 (0.62–1.76)	0.76 (0.51–1.13)	0.59 (0.38–0.93) *	0.72 (0.49–1.05)
Occupation not reported	1.60 (0.95–2.69)	0.97 (0.57–1.66)	0.95 (0.63–1.44)	0.67 (0.42–1.07)	0.82 (0.55–1.23)

* *p* < 0.05.

## Data Availability

The raw data supporting the conclusions of this article will be made available by the corresponding author on request.

## Supplementary Materials

The following supporting information can be downloaded at https://www.mdpi.com/article/10.3390/jcm15187027/s1, Supplementary File S1: post-transplant section of the survey questionnaire as administered; Supplementary File S2: STROBE checklist for cross-sectional studies.

## Abbreviations

The following abbreviations are used in this manuscript:

**Abbreviation**	**Meaning**
LT	Liver transplantation
QoL	Quality of life
HRQoL	Health-related quality of life
EU	European Union
UK	United Kingdom
ESOT	European Society for Organ Transplantation
PTG	Posttraumatic growth
GDPR	General Data Protection Regulation
PROM	Patient-Reported Outcome Measure
COSMIN	Consensus-based Standards for the selection of health Measurement Instruments
aOR	Adjusted odds ratio
CHERRIES	Checklist for Reporting Results of Internet E-Surveys
CI	Confidence interval
FDR	False discovery rate
IQR	Interquartile range
STROBE	Strengthening the Reporting of Observational Studies in Epidemiology
