# Supplementary material for: Quality of Life After Liver Transplantation—Results from a Large European Multinational Survey
Source: J Clin Med. 2026 Sep 10;15(18):7027. doi: 10.3390/jcm15187027 (PMC13607300; doi:10.3390/jcm15187027)
Supplement: Supplementary file 1 [file jcm-15-07027-s001.zip › jcm-4449030-supplementary.pdf]

Supplementary File S1 - Survey questionnaire (post-transplant section of the survey questionnaire as administered)

Supplementary File S1

Survey for the Quality of Life Before and After Liver Transplantation - section administered to liver transplant recipients

Manuscript jcm-4449030: "Quality of Life After Liver Transplantation - A Large European Multinational Survey"

Note. The instrument comprises two parallel sections, one for patients on the transplant waiting list and one for patients after transplantation. Only the section completed by transplant recipients is reproduced here, since only this section was used in the present analysis. The waiting-list section is not reported in this manuscript.

Respondent information

Type of transplant: ☐ kidney ☐ liver ☐ heart ☐ lung

☐ on the waiting list ☐ transplanted in …….. *(year of transplantation)*

This item served as the eligibility screening question. Only respondents who indicated a liver transplant and a year of transplantation were included in the present analysis; respondents reporting kidney, heart or lung transplantation were excluded from the present analysis.

Age: …………

Gender: …………

Place of residence: ……………………………………………

Level of education: ……………………………………………

Occupation: ……………………………………………

Marital status: ……………………………………………

How many people live with you? ☐ one ☐ two ☐ three ☐ four ☐ more than four

Survey for patients after liver transplantation

1. In general I would actually say my health is: Excellent · Very good · Good · Fair · Poor

2. Compared to one year ago, I would rate my health in general now: Much better than one year ago · Somewhat better than one year ago · About the same · Somewhat worse than one year ago · Much worse than one year ago

3. My health now limits me in physical activities such as moving a table, pushing a vacuum cleaner, climbing several flights of stairs: Yes, a lot · Yes, a little · Not at all

4. I find it hard to stand for long: Yes, a lot · Yes, a little · Not at all

5. My health now limits me in bathing or dressing myself: Yes, a lot · Yes, a little · Not at all

6. I need help to walk about outside: Yes, a lot · Yes, a little · Not at all

7. I need a caregiver for medication: ☐ Yes ☐ No

If yes:

8. I feel uncomfortable about depending on a caregiver for medication: ☐ Yes ☐ No

9. My physical health hinders my working and daily activities: Yes, a lot · Yes, a little · Not at all

10. I sleep badly at night: Yes, a lot · Yes, a little · Not at all

11. I have physical pain: Yes, a lot · Yes, a little · Not at all

12. I feel that life is worth living: Yes, a lot · Yes, a little · Not at all

13. I feel nervous: Yes, a lot · Yes, a little · Not at all

14. I feel free to plan a trip: Yes, a lot · Yes, a little · Not at all

15. I feel lonely: Yes, a lot · Yes, a little · Not at all

16. I find it difficult to reason and solve problems (for example making plans, making decisions, learning new things): Yes, a lot · Yes, a little · Not at all

17. I isolate myself as much as I can from the other people: Yes, a lot · Yes, a little · Not at all

18. I go out for entertainment (like meeting friends, going to the theatre or to the cinema, bowling, going to a concert, going to a sport match): More times in a week · At least once a week · More times in a month · At least once in a month · Less than once in a month

19. My social interaction changed after transplant: Yes, a lot · Yes, a little · Not at all

20. I am not doing the things I usually do to take care of my family: Yes, a lot · Yes, a little · Not at all

21. I feel able to stop or control worrying: Yes, a lot · Yes, a little · Not at all

22. I feel happy: Yes, a lot · Yes, a little · Not at all

23. I am generally satisfied with my sex life: Yes, a lot · Yes, a little · Not at all

24. I practise sport activities, such as walking, riding a bike, swimming or others: More times in a week · At least once a week · More times in a month · At least once in a month · Less than once in a month

25. My physical occurrence changed since I was transplanted and this has a positive impact on me: Yes, completely · Yes, partially · Very little · Not at all

26. I feel I've changed my life perception after transplant: Yes, a lot · Yes, a little · Not at all

27. I feel free to make plans for my future according to my wishes: Yes, a lot · Yes, a little · Not at all

28. I drink alcohol: Once a day · Once a week · Less than once a week · Never

29. I smoke cigarettes: More than a packet in a day · More than 10 in a day · More than 5 in a day · Never

30. I use drugs: Occasionally · Rarely · Never

31. I eat more than I should do or I eat unhealthy food: Occasionally · Rarely · Never

32. Before the transplant I have been on the transplant waiting list from: Less than one year · One year · More than one year

33. I have received appropriate information about liver transplantation by the medical staff: Yes, completely · Yes, partially · Very little · Not at all

34. I feel comfortable discussing my health issues with the medical and nurses staff: Yes, completely · Yes, partially · Very little · Not at all

35. I am satisfied with the information received about the therapies: Yes, completely · Yes, partially · Very little · Not at all

36. I take all the medications at the right time everyday: Always · Often · Sometimes · Rarely

37. After the transplant I've changed my habits about these behaviors:

	YES	NO
Drinking alcohol	☐	☐
Smoking cigarettes	☐	☐
Using drugs	☐	☐
Eating too much or eating unhealthy food	☐	☐

38. I would like to have more information about these issues:

	YES	NO
The side effects of the pharmacological therapies	☐	☐
Health limitations after liver transplantation	☐	☐
The impact of liver transplantation on my working activity	☐	☐
The impact of liver transplantation on performing regular sport activity	☐	☐
The impact of liver transplantation on sex life	☐	☐
Life expectation after liver transplantation	☐	☐

Supplementary File S2

STROBE Statement – Checklist of items that should be included in reports of cross-sectional studies

Manuscript: **jcm-4449030**

Section/topic	Item	STROBE recommendation	Reported in manuscript
Title and abstract	1	Indicate the study design with a commonly used term in the title or abstract; provide an informative and balanced summary of what was done and found.	Title; Abstract
Introduction – Background/rationale	2	Explain the scientific background and rationale for the investigation being reported.	Introduction
Introduction – Objectives	3	State specific objectives, including any prespecified hypotheses.	Introduction, final paragraph
Methods – Study design	4	Present key elements of study design early in the paper.	Section 2.1 Design
Methods – Setting	5	Describe the setting, locations, and relevant dates, including periods of recruitment, exposure, follow-up, and data collection.	Sections 2.1–2.2; recruitment period and European setting
Methods – Participants	6	Give eligibility criteria and the sources and methods of selection of participants.	Section 2.2 Participants
Methods – Variables	7	Clearly define outcomes, exposures, predictors, potential confounders, and effect modifiers; give diagnostic criteria if applicable.	Sections 2.3–2.4; Section 2.6 Statistical Analysis
Methods – Data sources/measurement	8	For each variable of interest, give sources of data and details of methods of assessment; describe comparability of assessment methods if more than one group is present.	Section 2.3 Survey Questionnaire; Supplementary File S1
Methods – Bias	9	Describe any efforts to address potential sources of bias.	Sections 2.2, 2.6; Section 4.6 Strengths and Limitations
Methods – Study size	10	Explain how the study size was arrived at.	Section 2.2 Participants; all eligible LT recipients completing the relevant survey section during the study period were included
Methods – Quantitative variables	11	Explain how quantitative variables were handled in the analyses; if applicable, describe groupings and why they were chosen.	Section 2.4 Definition of post-transplant duration groups; Section 2.6 Statistical Analysis
Methods – Statistical methods	12(a)	Describe all statistical methods, including those used to control for confounding.	Section 2.6 Statistical Analysis
Methods – Statistical methods	12(b)	Describe any methods used to examine subgroups and interactions.	Section 2.6; post-transplant duration group comparisons
Methods – Statistical methods	12(c)	Explain how missing data were addressed.	Section 2.6; item-specific denominators, missing responses not imputed
Methods – Statistical methods	12(d)	If applicable, describe analytical methods taking account of the sampling strategy.	Not applicable beyond the stated convenience-sampling design; recruitment approach described in Sections 2.2–2.3
Methods – Statistical methods	12(e)	Describe any sensitivity analyses.	Section 2.6 / not central to the primary analysis
Results – Participants	13(a)	Report numbers of individuals at each stage of the study.	Section 3 Results; Figure 1 participant flow
Results – Participants	13(b)	Give reasons for non-participation at each stage.	Section 2.2; Figure 1

Section/topic	Item	STROBE recommendation	Reported in manuscript
Results – Participants	13(c)	Consider use of a flow diagram.	Figure 1
Results – Descriptive data	14(a)	Give characteristics of study participants and information on exposures and potential confounders.	Table 1; Sections 3.1–3.2
Results – Descriptive data	14(b)	Indicate number of participants with missing data for each variable of interest.	Tables 1–2; item-specific denominators reported
Results – Outcome data	15	Report numbers of outcome events or summary measures.	Table 2; Sections 3.3–3.7
Results – Main results	16(a)	Give unadjusted estimates and, if applicable, confounder-adjusted estimates with precision; make clear which confounders were adjusted for and why.	Section 3.8; Table 4
Results – Main results	16(b)	Report category boundaries when continuous variables were categorized.	Section 2.4; Tables 1, 2 and 4
Results – Main results	16(c)	If relevant, consider translating estimates of relative risk into absolute risk for a meaningful time period.	Not applicable
Results – Other analyses	17	Report other analyses performed, including subgroup analyses, interactions, and sensitivity analyses.	Section 3.8; exploratory adjusted analyses for nominally significant items
Discussion – Key results	18	Summarize key results with reference to study objectives.	Discussion opening; Conclusions
Discussion – Limitations	19	Discuss limitations of the study, taking into account sources of potential bias or imprecision; discuss direction and magnitude where relevant.	Section 4.6 Strengths and Limitations
Discussion – Interpretation	20	Give a cautious overall interpretation considering objectives, limitations, multiplicity of analyses, results from similar studies, and other relevant evidence.	Sections 4.1–4.6
Discussion – Generalisability	21	Discuss the generalisability (external validity) of the study results.	Section 4.6 Strengths and Limitations
Other information – Funding	22	Give the source of funding and the role of the funders for the present study and, if applicable, for the original study on which the present article is based.	Funding statement
